# Supplementary material for: Evaluating competition for forage plants between honey bees and wild bees in Denmark
Source: PLoS One. 2021 Apr 28;16(4):e0250056. doi: 10.1371/journal.pone.0250056 (PMC8081269; doi:10.1371/journal.pone.0250056)
Supplement: S1 Table — While the Bombus lucorum-complex in Denmark is near impossible to distinguish morphologically, in particular amongst the workers, they are here maintained separately because of certain known forage-differences [118]. Andrena albofasciata is considered a junior synonym of A. ovatula by [63, 64], but retained here in agreement with [57]. Lecty is not fully confirmed for Andrena nanula and Lasioglossum sexnotatulum. The former visits Compositae and Rosaceae in Denmark, although that might have been nectar sources, as it is suspected oligolectic on Apiaceae in neighboring countries [119]. The latter species is suspected polylectic based on forage plants in Finland [120]. Other species may be to strictly defined by Scheuchl and Willner [63], e.g. the polylectic Andrena ovatula, Colletes cunicularis, Hoplitis leucomelana, Megachile circumcincta and M. lagopoda which at least have specific pollen preferences [e.g., 121], and might be considered oligolectic. (PDF) [file pone.0250056.s001.pdf]

S1 Table. List of Danish species of bees, both wild and honey bees; foraging specialization [based on 1]; Danish Red List category [based on 2]; status as threatened (VU+EN+CR), not threatened (LC+NT), other (NA+DD) or regionally extinct (RE); total number of known forage plants (FP) for the bee species (0 when forage plants are unknown in the revised literature); number of the forage plants only visited by the wild bee species, but not by honey bees; overlap of forage plants that are visited both by wild bee species and by honey bees; MacArthur and Levins asymmetrical measure for niche overlap of honey bee species on wild bee species. While the *Bombus lucorum*-complex in Denmark is near impossible to distinguish morphologically, in particular amongst the workers, they are here maintained separately because of certain known forage-differences [3]. *Andrena albofasciata* is considered a junior synonym of *A. ovatula* by [1, 4], but retained here in agreement with [2]. Lecty is not fully confirmed for *Andrena nanula* and *Lasioglossum sexnotatulum*. The former visits Compositae and Rosaceae in Denmark, although that might have been nectar sources, as it is suspected oligolectic on Apiaceae in neighboring countries [5]. The latter species is suspected polylectic based on forage plants in Finland [6]. Other species may be to strictly defined by Scheuchl and Willner [1], e.g. the polylectic *Andrena ovatula*, *Colletes cunicularis*, *Hoplitis leucomelana*, *Megachile circumcincta* and *M. lagopoda* which at least have specific pollen preferences [e.g., 7], and might be considered oligolectic.

Species	Lecty	DK Red List	Threatened	Total forage plants (FP)	FP without overlap	FP overlap	MacArthur & Levins (%)
Andrena albofasciata	Polylectic	VU	Threatened	0	0	0	0.0
Andrena alfkenella	Polylectic	CR	Threatened	20	5	15	75.0
Andrena angustior	Polylectic	DD	other	14	2	12	85.7
Andrena apicata	Oligolectic	LC	Not threatened	5	0	5	100.0
Andrena argentata	Polylectic	EN	Threatened	14	2	12	85.7
Andrena barbilabris	Polylectic	LC	Not threatened	22	2	20	90.9
Andrena bicolor	Polylectic	LC	Not threatened	42	10	32	76.2
Andrena bimaculata	Polylectic	NT	Not threatened	12	4	8	66.7
Andrena carantonica	Polylectic	LC	Not threatened	18	1	17	94.4
Andrena chrysopyga	Polylectic	VU	Threatened	13	3	10	76.9
Andrena chrysosceles	Polylectic	LC	Not threatened	28	7	21	75.0
Andrena cineraria	Polylectic	LC	Not threatened	20	5	15	75.0
Andrena clarkella	Oligolectic	LC	Not threatened	3	0	3	100.0
Andrena coitana	Polylectic	EN	Threatened	21	9	12	57.1
Andrena curvungula	Oligolectic	NA	other	2	1	1	50.0
Andrena denticulata	Oligolectic	LC	Not threatened	13	7	6	46.2
Andrena falsifica	Polylectic	NA	other	6	0	6	100.0

Andrena flavipes	Polylectic	LC	Not threatened	58	21	37	63.8
Andrena fucata	Polylectic	LC	Not threatened	19	2	17	89.5
Andrena fulva	Polylectic	LC	Not threatened	21	1	20	95.2
Andrena fulvago	Oligolectic	VU	Threatened	10	6	4	40.0
Andrena fulvida	Polylectic	NT	Not threatened	18	0	18	100.0
Andrena fuscipes	Oligolectic	LC	Not threatened	1	0	1	100.0
Andrena gelriae	Oligolectic	RE	(extinct)	3	0	3	100.0
Andrena gravida	Polylectic	NT	Not threatened	10	2	8	80.0
Andrena haemorrhoa	Polylectic	LC	Not threatened	39	6	33	84.6
Andrena hattorfiana	Oligolectic	LC	Not threatened	6	1	5	83.3
Andrena helvola	Polylectic	LC	Not threatened	12	0	12	100.0
Andrena humilis	Oligolectic	NT	Not threatened	7	5	2	28.6
Andrena intermedia	Oligolectic	NA	other	2	0	2	100.0
Andrena labialis	Oligolectic	LC	Not threatened	6	2	4	66.7
Andrena labiata	Polylectic	LC	Not threatened	21	4	17	81.0
Andrena lapponica	Oligolectic	LC	Not threatened	8	0	8	100.0
Andrena lathyri	Oligolectic	VU	Threatened	2	0	2	100.0
Andrena marginata	Oligolectic	EN	Threatened	8	1	7	87.5
Andrena minutula	Polylectic	LC	Not threatened	37	9	28	75.7
Andrena minutuloides	Polylectic	DD	other	28	5	23	82.1
Andrena morawitzi	Polylectic	EN	Threatened	0	0	0	0.0
Andrena nanula	Polylectic	NA	other	3	1	2	66.7
Andrena nasuta	Oligolectic	NA	other	1	0	1	100.0
Andrena nigriceps	Polylectic	LC	Not threatened	17	6	11	64.7
Andrena nigroaenea	Polylectic	LC	Not threatened	29	8	21	72.4
Andrena nigrospina	Polylectic	NT	Not threatened	8	3	5	62.5
Andrena nitida	Polylectic	NT	Not threatened	31	4	27	87.1
Andrena niveata	Oligolectic	RE	(extinct)	8	2	6	75.0
Andrena nycthemera	Oligolectic	NA	other	1	0	1	100.0
Andrena ovatula	Polylectic	VU	Threatened	11	1	10	90.9
Andrena praecox	Oligolectic	LC	Not threatened	1	0	1	100.0

Andrena proxima	Oligolectic	NA	other	13	6	7	53.8
Andrena ruficrus	Oligolectic	LC	Not threatened	2	0	2	100.0
Andrena schencki	Polylectic	RE	(extinct)	8	1	7	87.5
Andrena semilaevis	Polylectic	LC	Not threatened	4	0	4	100.0
Andrena similis	Oligolectic	DD	other	8	2	6	75.0
Andrena simillima	Polylectic	NA	other	6	2	4	66.7
Andrena subopaca	Polylectic	LC	Not threatened	8	2	6	75.0
Andrena synadelpha	Polylectic	DD	other	14	1	13	92.9
Andrena tarsata	Oligolectic	NT	Not threatened	7	1	6	85.7
Andrena thoracica	Polylectic	EN	Threatened	24	7	17	70.8
Andrena tibialis	Polylectic	LC	Not threatened	12	0	12	100.0
Andrena vaga	Oligolectic	LC	Not threatened	1	0	1	100.0
Andrena varians	Polylectic	NT	Not threatened	19	1	18	94.7
Andrena viridescens	Oligolectic	NA	other	1	0	1	100.0
Andrena wilkella	Oligolectic	LC	Not threatened	11	1	10	90.9
Anthidiellum strigatum	Polylectic	NA	other	8	1	7	87.5
Anthidium manicatum	Polylectic	LC	Not threatened	25	11	14	56.0
Anthidium punctatum	Polylectic	LC	Not threatened	7	1	6	85.7
Anthophora aestivalis	Polylectic	CR	Threatened	10	3	7	70.0
Anthophora bimaculata	Polylectic	NA	other	23	6	17	73.9
Anthophora furcata	Oligolectic	LC	Not threatened	12	3	9	75.0
Anthophora plagiata	Polylectic	RE	(extinct)	7	1	6	85.7
Anthophora plumipes	Polylectic	LC	Not threatened	26	5	21	80.8
Anthophora quadrimaculata	Polylectic	LC	Not threatened	15	4	11	73.3
Anthophora retusa	Polylectic	EN	Threatened	14	5	9	64.3
Apis mellifera	Polylectic	NA	other	294	0	294	100.0
Biastes truncatus	[Parasite]	CR	Threatened	1	0	1	100.0
Bombus barbutellus	[Parasite]	EN	Threatened	12	3	9	75.0
Bombus bohemicus	[Parasite]	LC	Not threatened	10	1	9	90.0
Bombus campestris	[Parasite]	LC	Not threatened	11	2	9	81.8
Bombus cryptarum	Polylectic	LC	Not threatened	7	1	6	85.7

Bombus cullumanus	Polylectic	RE	(extinct)	5	1	4	80.0
Bombus distinguendus	Polylectic	CR	Threatened	17	4	13	76.5
Bombus hortorum	Polylectic	LC	Not threatened	21	6	15	71.4
Bombus humilis	Polylectic	NT	Not threatened	20	8	12	60.0
Bombus hypnorum	Polylectic	LC	Not threatened	9	0	9	100.0
Bombus jonellus	Polylectic	LC	Not threatened	9	2	7	77.8
Bombus lapidarius	Polylectic	LC	Not threatened	15	3	12	80.0
Bombus lucorum	Polylectic	LC	Not threatened	13	3	10	76.9
Bombus magnus	Polylectic	LC	Not threatened	7	2	5	71.4
Bombus muscorum	Polylectic	LC	Not threatened	16	5	11	68.8
Bombus norvegicus	[Parasite]	LC	Not threatened	7	1	6	85.7
Bombus pascuorum	Polylectic	LC	Not threatened	20	6	14	70.0
Bombus pomorum	Polylectic	RE	(extinct)	2	0	2	100.0
Bombus pratorum	Polylectic	LC	Not threatened	13	0	13	100.0
Bombus quadricolor	[Parasite]	RE	(extinct)	0	0	0	0.0
Bombus ruderarius	Polylectic	NT	Not threatened	10	3	7	70.0
Bombus ruderatus	Polylectic	RE	(extinct)	13	2	11	84.6
Bombus rupestris	[Parasite]	LC	Not threatened	17	5	12	70.6
Bombus soroeensis	Polylectic	LC	Not threatened	11	0	11	100.0
Bombus subterraneus	Polylectic	NT	Not threatened	7	0	7	100.0
Bombus sylvarum	Polylectic	EN	Threatened	12	5	7	58.3
Bombus sylvestris	[Parasite]	LC	Not threatened	11	1	10	90.9
Bombus terrestris	Polylectic	LC	Not threatened	18	4	14	77.8
Bombus vestalis	[Parasite]	LC	Not threatened	11	3	8	72.7
Bombus veteranus	Polylectic	EN	Threatened	0	0	0	0.0
Chelostoma campanularum	Oligolectic	LC	Not threatened	5	3	2	40.0
Chelostoma florisomne	Oligolectic	LC	Not threatened	5	0	5	100.0
Chelostoma rapunculi	Oligolectic	LC	Not threatened	1	0	1	100.0
Coelioxys conica	[Parasite]	LC	Not threatened	2	1	1	50.0
Coelioxys conoidea	[Parasite]	EN	Threatened	18	4	14	77.8
Coelioxys elongata	[Parasite]	LC	Not threatened	10	2	8	80.0

Coelioxys inermis	[Parasite]	LC	Not threatened	7	0	7	100.0
Coelioxys mandibularis	[Parasite]	LC	Not threatened	11	0	11	100.0
Coelioxys rufescens	[Parasite]	NT	Not threatened	18	3	15	83.3
Colletes cunicularius	Polylectic	LC	Not threatened	9	1	8	88.9
Colletes daviesanus	Oligolectic	LC	Not threatened	11	10	1	9.1
Colletes floralis	Polylectic	CR	Threatened	16	6	10	62.5
Colletes fodiens	Oligolectic	LC	Not threatened	9	8	1	11.1
Colletes halophilus	Oligolectic	DD	other	6	5	1	16.7
Colletes impunctatus	Polylectic	NT	Not threatened	1	0	1	100.0
Colletes marginatus	Oligolectic	NT	Not threatened	5	0	5	100.0
Colletes similis	Oligolectic	LC	Not threatened	10	8	2	20.0
Colletes succinctus	Oligolectic	LC	Not threatened	8	2	6	75.0
Dasypoda hirtipes	Oligolectic	LC	Not threatened	13	10	3	23.1
Dasypoda suripes	Oligolectic	CR	Threatened	1	0	1	100.0
Dufourea dentiventris	Oligolectic	EN	Threatened	2	1	1	50.0
Dufourea halictula	Oligolectic	EN	Threatened	1	0	1	100.0
Dufourea inermis	Oligolectic	EN	Threatened	1	0	1	100.0
Dufourea minuta	Oligolectic	RE	(extinct)	6	3	3	50.0
Epeoloides coecutiens	[Parasite]	NA	other	9	1	8	88.9
Epeolus alpinus	[Parasite]	NT	Not threatened	7	1	6	85.7
Epeolus cruciger	[Parasite]	LC	Not threatened	5	2	3	60.0
Epeolus variegatus	[Parasite]	LC	Not threatened	8	5	3	37.5
Eucera longicornis	Oligolectic	LC	Not threatened	8	2	6	75.0
Halictus compressus	Polylectic	RE	(extinct)	3	0	3	100.0
Halictus confusus	Polylectic	LC	Not threatened	13	1	12	92.3
Halictus leucaheneus	Polylectic	CR	Threatened	15	4	11	73.3
Halictus maculatus	Polylectic	CR	Threatened	27	11	16	59.3
Halictus quadricinctus	Polylectic	VU	Threatened	19	6	13	68.4
Halictus rubicundus	Polylectic	LC	Not threatened	29	8	21	72.4
Halictus sexcinctus	Polylectic	NA	other	14	5	9	64.3
Halictus tumulorum	Polylectic	LC	Not threatened	37	11	26	70.3

Heriades truncorum	Oligolectic	NT	Not threatened	25	16	9	36.0
Hoplitis adunca	Oligolectic	NA	other	1	0	1	100.0
Hoplitis anthocopoides	Oligolectic	VU	Threatened	1	0	1	100.0
Hoplitis claviventris	Polylectic	LC	Not threatened	17	3	14	82.4
Hoplitis leucomelana	Polylectic	NA	other	10	1	9	90.0
Hoplosmia spinulosa	Oligolectic	VU	Threatened	23	14	9	39.1
Hylaeus angustatus	Polylectic	NA	other	12	2	10	83.3
Hylaeus brevicornis	Polylectic	LC	Not threatened	12	3	9	75.0
Hylaeus clypearis	Polylectic	NA	other	7	1	6	85.7
Hylaeus communis	Polylectic	LC	Not threatened	17	4	13	76.5
Hylaeus confusus	Polylectic	LC	Not threatened	6	0	6	100.0
Hylaeus cornutus	Polylectic	NA	other	10	2	8	80.0
Hylaeus difformis	Polylectic	NA	other	9	0	9	100.0
Hylaeus dilatatus	Polylectic	LC	Not threatened	14	4	10	71.4
Hylaeus gracilicornis	Polylectic	NA	other	0	0	0	0.0
Hylaeus hyalinatus	Polylectic	LC	Not threatened	13	5	8	61.5
Hylaeus incongruus	Polylectic	LC	Not threatened	19	5	14	73.7
Hylaeus pectoralis	Polylectic	LC	Not threatened	8	2	6	75.0
Hylaeus pfankuchi	Polylectic	RE	(extinct)	0	0	0	0.0
Hylaeus pictipes	Polylectic	CR	Threatened	12	3	9	75.0
Hylaeus punctulatissimus	Oligolectic	NA	other	3	0	3	100.0
Hylaeus rinki	Polylectic	RE	(extinct)	5	1	4	80.0
Hylaeus signatus	Oligolectic	DD	other	3	1	2	66.7
Hylaeus sinuatus	Polylectic	RE	(extinct)	6	1	5	83.3
Hylaeus variegatus	Polylectic	NA	other	16	4	12	75.0
Lasioglossum aeratum	Polylectic	CR	Threatened	4	0	4	100.0
Lasioglossum albipes	Polylectic	LC	Not threatened	25	6	19	76.0
Lasioglossum brevicorne	Polylectic	VU	Threatened	5	3	2	40.0
Lasioglossum calceatum	Polylectic	LC	Not threatened	46	15	31	67.4
Lasioglossum costulatum	Oligolectic	NA	other	2	0	2	100.0
Lasioglossum fratellum	Polylectic	LC	Not threatened	13	3	10	76.9

Lasioglossum fulvicorne	Polylectic	VU	Threatened	11	2	9	81.8
Lasioglossum laevigatum	Polylectic	NA	other	23	5	18	78.3
Lasioglossum lativentre	Polylectic	NT	Not threatened	6	0	6	100.0
Lasioglossum leucopus	Polylectic	LC	Not threatened	3	0	3	100.0
Lasioglossum leucozonium	Polylectic	LC	Not threatened	31	12	19	61.3
Lasioglossum lucidulum	Polylectic	LC	Not threatened	7	0	7	100.0
Lasioglossum malachurum	Polylectic	NA	other	31	8	23	74.2
Lasioglossum minutissimum	Polylectic	LC	Not threatened	5	1	4	80.0
Lasioglossum morio	Polylectic	LC	Not threatened	26	6	20	76.9
Lasioglossum nitidiusculum	Polylectic	NT	Not threatened	15	3	12	80.0
Lasioglossum nitidulum	Polylectic	VU	Threatened	28	9	19	67.9
Lasioglossum parvulum	Polylectic	LC	Not threatened	9	2	7	77.8
Lasioglossum pauxillum	Polylectic	NA	other	37	15	22	59.5
Lasioglossum punctatissimum	Polylectic	LC	Not threatened	14	3	11	78.6
Lasioglossum quadrinotatum	Polylectic	LC	Not threatened	7	2	5	71.4
Lasioglossum rufitarse	Polylectic	LC	Not threatened	7	3	4	57.1
Lasioglossum semilucens	Polylectic	LC	Not threatened	1	0	1	100.0
Lasioglossum sexmaculatum	Polylectic	NT	Not threatened	0	0	0	0.0
Lasioglossum sexnotatulum	Polylectic	NA	other	0	0	0	0.0
Lasioglossum sexnotatum	Polylectic	RE	(extinct)	19	2	17	89.5
Lasioglossum sexstrigatum	Polylectic	LC	Not threatened	8	3	5	62.5
Lasioglossum tarsatum	Polylectic	NT	Not threatened	4	1	3	75.0
Lasioglossum villosulum	Polylectic	LC	Not threatened	18	7	11	61.1
Lasioglossum xanthopus	Polylectic	VU	Threatened	18	6	12	66.7
Lasioglossum zonulum	Polylectic	EN	Threatened	23	7	16	69.6
Macropis europaea	Oligolectic	LC	Not threatened	9	2	7	77.8
Macropis fulvipes	Oligolectic	NA	other	4	1	3	75.0
Megachile alpicola	Polylectic	NA	other	7	2	5	71.4
Megachile analis	Polylectic	NT	Not threatened	5	0	5	100.0
Megachile apicalis	Polylectic	NA	other	5	1	4	80.0
Megachile centuncularis	Polylectic	LC	Not threatened	20	11	9	45.0

Megachile circumcincta	Polylectic	LC	Not threatened	17	6	11	64.7
Megachile lagopoda	Polylectic	LC	Not threatened	11	4	7	63.6
Megachile lapponica	Oligolectic	LC	Not threatened	3	0	3	100.0
Megachile leachella	Polylectic	LC	Not threatened	12	3	9	75.0
Megachile maritima	Polylectic	VU	Threatened	18	6	12	66.7
Megachile nigriventris	Oligolectic	NA	other	6	1	5	83.3
Megachile rotundata	Polylectic	NA	other	9	2	7	77.8
Megachile versicolor	Polylectic	LC	Not threatened	14	4	10	71.4
Megachile willughbiella	Polylectic	LC	Not threatened	16	4	12	75.0
Melecta albifrons	[Parasite]	LC	Not threatened	11	1	10	90.9
Melecta luctuosa	[Parasite]	RE	(extinct)	7	1	6	85.7
Melitta haemorrhoidalis	Oligolectic	LC	Not threatened	5	1	4	80.0
Melitta leporina	Oligolectic	LC	Not threatened	8	2	6	75.0
Melitta nigricans	Oligolectic	NA	other	1	0	1	100.0
Melitta tricincta	Oligolectic	VU	Threatened	1	1	0	0.0
Nomada alboguttata	[Parasite]	NT	Not threatened	5	0	5	100.0
Nomada argentata	[Parasite]	RE	(extinct)	3	1	2	66.7
Nomada armata	[Parasite]	NT	Not threatened	2	0	2	100.0
Nomada baccata	[Parasite]	EN	Threatened	5	2	3	60.0
Nomada distinguenda	[Parasite]	NA	other	5	2	3	60.0
Nomada fabriciana	[Parasite]	LC	Not threatened	14	4	10	71.4
Nomada ferruginata	[Parasite]	LC	Not threatened	6	0	6	100.0
Nomada flava	[Parasite]	LC	Not threatened	11	3	8	72.7
Nomada flavoguttata	[Parasite]	LC	Not threatened	12	0	12	100.0
Nomada flavopicta	[Parasite]	LC	Not threatened	15	5	10	66.7
Nomada fucata	[Parasite]	LC	Not threatened	8	1	7	87.5
Nomada fulvicornis	[Parasite]	LC	Not threatened	10	2	8	80.0
Nomada fuscicornis	[Parasite]	EN	Threatened	6	4	2	33.3
Nomada goodeniana	[Parasite]	LC	Not threatened	8	1	7	87.5
Nomada guttulata	[Parasite]	CR	Threatened	4	1	3	75.0
Nomada integra	[Parasite]	CR	Threatened	7	2	5	71.4

Nomada lathburiana	[Parasite]	LC	Not threatened	6	1	5	83.3
Nomada leucophthalma	[Parasite]	LC	Not threatened	6	0	6	100.0
Nomada marshamella	[Parasite]	LC	Not threatened	5	0	5	100.0
Nomada moeschleri	[Parasite]	VU	Threatened	0	0	0	0.0
Nomada mutabilis	[Parasite]	NA	other	4	2	2	50.0
Nomada obscura	[Parasite]	CR	Threatened	3	0	3	100.0
Nomada obtusifrons	[Parasite]	CR	Threatened	11	2	9	81.8
Nomada opaca	[Parasite]	RE	(extinct)	1	0	1	100.0
Nomada panzeri	[Parasite]	LC	Not threatened	11	1	10	90.9
Nomada roberjeotiana	[Parasite]	EN	Threatened	11	3	8	72.7
Nomada ruficornis	[Parasite]	LC	Not threatened	3	1	2	66.7
Nomada rufipes	[Parasite]	LC	Not threatened	9	2	7	77.8
Nomada sheppardana	[Parasite]	LC	Not threatened	4	2	2	50.0
Nomada signata	[Parasite]	LC	Not threatened	7	1	6	85.7
Nomada similis	[Parasite]	NT	Not threatened	5	3	2	40.0
Nomada stigma	[Parasite]	VU	Threatened	4	1	3	75.0
Nomada striata	[Parasite]	NT	Not threatened	16	3	13	81.3
Nomada succincta	[Parasite]	CR	Threatened	3	0	3	100.0
Nomada villosa	[Parasite]	RE	(extinct)	2	0	2	100.0
Osmia aurulenta	Polylectic	LC	Not threatened	33	9	24	72.7
Osmia bicornis	Polylectic	LC	Not threatened	35	4	31	88.6
Osmia brevicornis	Oligolectic	NA	other	7	2	5	71.4
Osmia caerulescens	Polylectic	LC	Not threatened	26	7	19	73.1
Osmia cornuta	Polylectic	NA	other	19	2	17	89.5
Osmia leaiana	Oligolectic	LC	Not threatened	16	9	7	43.8
Osmia maritima	Polylectic	NT	Not threatened	7	2	5	71.4
Osmia niveata	Oligolectic	CR	Threatened	6	2	4	66.7
Osmia parietina	Polylectic	NA	other	8	1	7	87.5
Osmia pilicornis	Polylectic	CR	Threatened	13	4	9	69.2
Osmia uncinata	Polylectic	VU	Threatened	19	3	16	84.2
Osmia xanthomelana	Oligolectic	NA	other	2	1	1	50.0

Panurgus banksianus	Oligolectic	LC	Not threatened	8	5	3	37.5
Panurgus calcaratus	Oligolectic	LC	Not threatened	8	6	2	25.0
Rophites quinquespinosus	Oligolectic	RE	(extinct)	5	2	3	60.0
Sphecodes albilabris	[Parasite]	LC	Not threatened	10	5	5	50.0
Sphecodes crassus	[Parasite]	LC	Not threatened	12	3	9	75.0
Sphecodes ephippius	[Parasite]	LC	Not threatened	14	2	12	85.7
Sphecodes ferruginatus	[Parasite]	CR	Threatened	3	0	3	100.0
Sphecodes geoffrellus	[Parasite]	LC	Not threatened	11	2	9	81.8
Sphecodes gibbus	[Parasite]	LC	Not threatened	23	5	18	78.3
Sphecodes longulus	[Parasite]	NA	other	4	1	3	75.0
Sphecodes marginatus	[Parasite]	NA	other	5	2	3	60.0
Sphecodes miniatus	[Parasite]	LC	Not threatened	5	2	3	60.0
Sphecodes monilicornis	[Parasite]	LC	Not threatened	10	3	7	70.0
Sphecodes niger	[Parasite]	VU	Threatened	3	1	2	66.7
Sphecodes pellucidus	[Parasite]	LC	Not threatened	11	4	7	63.6
Sphecodes puncticeps	[Parasite]	LC	Not threatened	8	1	7	87.5
Sphecodes reticulatus	[Parasite]	LC	Not threatened	6	4	2	33.3
Sphecodes rubicundus	[Parasite]	VU	Threatened	5	1	4	80.0
Sphecodes rufiventris	[Parasite]	NA	other	0	0	0	0.0
Sphecodes spinulosus	[Parasite]	NA	other	5	0	5	100.0
Stelis breviuscula	[Parasite]	NA	other	15	10	5	33.3
Stelis minuta	[Parasite]	NA	other	3	0	3	100.0
Stelis ornatula	[Parasite]	LC	Not threatened	11	6	5	45.5
Stelis phaeoptera	[Parasite]	VU	Threatened	11	5	6	54.5
Stelis punctulatissima	[Parasite]	VU	Threatened	21	6	15	71.4
Trachusa byssina	Oligolectic	NA	other	7	2	5	71.4
Xylocopa violacea	Polylectic	NA	other	12	1	11	91.7

References:

1. Scheuchl E, Willner W. Taschenlexikon der Wildbienen Mitteleuropas. Alle Arten im Porträt. Wiebelsheim: Quelle & Meyer; 2016. 917 p.
2. Madsen HB. Bier. In: Moeslund JE, Nygaard B, Erjnæs R, Bell N, Bruun LD, Bygebjerg R, et al., editors. Den danske Rødliste 2019. Aarhus: Aarhus Universitet, DCE – Nationalt Center for Miljø og Energi; 2019.
3. Bertsch A, Schweer H, Titze A. Discrimination of the bumblebee species *Bombus lucorum, B. cryptarum* and *B. magnus* by morphological characters and male labial gland secretions (Hymenoptera: Apidae). Beiträge zur Entomologie. 2004;54(2):365–86.
4. Westrich P. Die Wildbienen Deutschlands. Stuttgart: Ulmer Verlag; 2018.
5. Madsen HB, Poulsen KR, Rasmussen C, Calabuig I, Schmidt HT. Fire bier nye for den danske fauna (Hymenoptera, Apoidea, Apiformes). Entomologiske Meddelelser. 2018;86(1-2):39–50.
6. Söderman G, Leinonen R. Suomen mesipistiäiset ja niiden uhanalaisuus. Helsinki: Tremex Press Oy; 2003. 420 p.
7. Bischoff I, Feltgen K, Breckner D. Foraging strategy and pollen preferences of *Andrena vaga* (Panzer) and *Colletes cunicularius* (L.) (Hymenoptera: Apidae). Journal of Hymenoptera Research. 2003;12:220–37.
