# Supplementary material for: Evaluating competition for forage plants between honey bees and wild bees in Denmark
Source: PLoS One. 2021 Apr 28;16(4):e0250056. doi: 10.1371/journal.pone.0250056 (PMC8081269; doi:10.1371/journal.pone.0250056)
Supplement: Attachment — Submitted filename: Response to Reviewers.docx [file pone.0250056.s004.docx]

Response to Reviewers letter which should include a point by point response to each of the points made by the Editor and / or Reviewers.

2. Please review your reference list to ensure that it is complete and correct. If you have cited papers that have been retracted, please include the rationale for doing so in the manuscript text, or remove these references and replace them with relevant current references. Any changes to the reference list should be mentioned in the rebuttal letter that accompanies your revised manuscript. If you need to cite a retracted article, indicate the article’s retracted status in the References list and also include a citation and full reference for the retraction notice.

AUTHOR: Yes, it has been revised and no known retracted papers are cited.

3. Please remove your figures from within your manuscript file, leaving only the individual TIFF/EPS image files, uploaded separately. These will be automatically included in the reviewers’ PDF.

AUTHOR: Yes, all removed and uploaded.

1. Is the manuscript technically sound, and do the data support the conclusions?

The manuscript must describe a technically sound piece of scientific research with data that supports the conclusions. Experiments must have been conducted rigorously, with appropriate controls, replication, and sample sizes. The conclusions must be drawn appropriately based on the data presented.

Reviewer #1: Yes

Reviewer #2: Yes

AUTHOR: OK

2. Has the statistical analysis been performed appropriately and rigorously?

Reviewer #1: Yes

Reviewer #2: Yes

AUTHOR: OK

3. Have the authors made all data underlying the findings in their manuscript fully available?

The PLOS Data policy requires authors to make all data underlying the findings described in their manuscript fully available without restriction, with rare exception (please refer to the Data Availability Statement in the manuscript PDF file). The data should be provided as part of the manuscript or its supporting information, or deposited to a public repository. For example, in addition to summary statistics, the data points behind means, medians and variance measures should be available. If there are restrictions on publicly sharing data—e.g. participant privacy or use of data from a third party—those must be specified.

Reviewer #1: Yes

Reviewer #2: Yes

AUTHOR: OK

4. Is the manuscript presented in an intelligible fashion and written in standard English?

PLOS ONE does not copyedit accepted manuscripts, so the language in submitted articles must be clear, correct, and unambiguous. Any typographical or grammatical errors should be corrected at revision, so please note any specific errors here.

Reviewer #1: Yes

Reviewer #2: Yes

AUTHOR: OK

5. Review Comments to the Author

Please use the space provided to explain your answers to the questions above. You may also include additional comments for the author, including concerns about dual publication, research ethics, or publication ethics. (Please upload your review as an attachment if it exceeds 20,000 characters)

Reviewer #1: I have reviewed the manuscript entitled "Evaluating competition for forage plants between honey bees and wild bees in Denmark". This is an interesting approach based on data available in the literature, which investigated floral resources shared by honeybees and wild bees, with an emphasis on both threatened wild bee species and foraging specialist species from Denmark. It is an original and very well written paper investigating the potential for food competition among wild bees and honeybees. The authors based their analysis on data available for 292 bee species. The conservation status of these species was also considered. I have two main concerns: i) the list of plants used by the 292 Danish bee species was restricted to plant genus, which would not be the most desirable for the best conclusion; ii) for wild bees, the authors extracted information on all forage plants from neighboring German species. Despite this, I still believe that the study is innovative and the results are relevant. Thus, I suggest accepting this paper after minor revision.

AUTHOR: Both concerns were addressed in methods and discussion. Local data are not in existence.

P.2 – L.70: to insufficient, or a reduced quality of, pollination – delete the comma changing to: to insufficient or a reduced quality of pollination

AUTHOR: Corrected.

P. 2- L.75-76: Please give the proper credit to Henry and Rodet 2018 (Ref [46]). The idea in lines 75-76 was sustained by the study of these authors. Please, give them the credit.

AUTHOR: Added.

P.6 – L.267: replace plans by plants

AUTHOR: Corrected.

P.6 – L. 269-270: give emphasis to the negative association (rs) between the total number of food plants and pairwise niche overlap.

Figure 1 legend: polynomial trend line (R² = 0,1499) – replace comma by dot: 0.1499

AUTHOR: Added.

Reviewer #2: This is a very interesting and elegant study evaluating competition between (managed) honey bees and wild bees over floral resources in Denmark. I applaud the aim of this study and using an impressive database, the authors analyse the data in a creative way, thereby providing useful guidelines for conservation efforts. The manuscript is beautifully written, clear and precise and the data properly analysed. I therefore highly recommend publication and I only have a few minor remarks.

L41: Consider changing: the word ‘network’ with ‘interactions’

AUTHOR: Corrected.

L45: I would replace the abbreviations ‘VU+EN+CR’ by full names

AUTHOR: Corrected.

L86: I think the references here should include ‘Santos et al., 2012’; or ‘Santos et al., 2012’ should replace reference ‘42’

AUTHOR: Added.

L89: I suggest to rephrase to ‘depressing species richness (Angelella et al., 2021) and local populations’

AUTHOR: Added.

L135: ‘honey’ should read ‘nectar’

AUTHOR: Corrected.

L220-222: Consider to include these two sentences in Abstract. It is impressive that honey bees and wild bees share 176 plant genera in Denmark (almost 43% of known plant genera recorded as forage plants)

AUTHOR: 176 plant genera in Denmark was added.

L266: Please, consider using “hereafter referred to as K-W’

AUTHOR: I did not follow as it was only in a very few instances and would not save much space.

L268, Appendix 1 (8th column): Replace comma by dot

AUTHOR: Corrected.

L327: ‘nearby bee’ should read ‘nearby honey bee’

AUTHOR: Corrected.

L340: ‘figure 5a’ should read ‘Figure 5a’

AUTHOR: Corrected.

Fig. 1: Since there are 5 figures and 1 table, I would transfer this figure to ‘Supporting Information’ section

AUTHOR: I believe it is of value and have left it to be placed in the text.

Figs. 1, 2, 3: Please, add axis titles

AUTHOR: Corrected.

Fig. 2: I would replace the abbreviations by full names and add ‘Red list categories’ to the legend

AUTHOR: Corrected.

Fig. 4: ‘plant genus’ should read ‘plant genera’ and ‘No Plant’ should read ‘No plant’

AUTHOR: Corrected.

Fig. 5: I ‘redrew’ this figure (please find the attached file for your consideration)

AUTHOR: Thank you. We used this for inspiration and redraw the figure.

References

Angelella G, McCullough C, O’Rourke M (2021). Honey bee hives decrease wild bee abundance, species richness, and fruit count on farms regardless of wildflower strips. Scientific reports 11, 3202.

Santos GMM, Aguiar CM, Genini J, Martins CF, Zanella FC, Mello MA (2012). Invasive Africanized honeybees change the structure of native pollination networks in Brazil. Biological Invasions 14, 2369-2378.

AUTHOR: Added.
